# Fabrication of hafnium-based nanoparticles and nanostructures using picosecond laser ablation

**DOI:** 10.3762/bjnano.15.129

**Published:** 2024-12-18

**Authors:** Abhishek Das, Mangababu Akkanaboina, Jagannath Rathod, R Sai Prasad Goud, Kanaka Ravi Kumar, Raghu C Reddy, Ratheesh Ravendran, Katia Vutova, S V S Nageswara Rao, Venugopal Rao Soma

**Affiliations:** 1 School of Physics, University of Hyderabad, Hyderabad 500046, Telangana, India; 2 Department of Physics, Banaras Hindu University, Varanasi 221005, Uttar Pradesh, India; 3 Advanced Centre of Research in High Energy Materials (ACRHEM), DRDO Industry Academia – Centre of Excellence (DIA-COE), University of Hyderabad, Hyderabad 500046, Telangana, India; 4 Centre for Materials for Electronics Technology (C-MET), IDA Phase III, Cherlapalli, HCL (P.O.), Hyderabad 500 051, Telangana, India; 5 Institute of Electronics, Bulgarian Academy of Sciences, 72, Tzarigradsko Shosse, 1784, Sofia, Bulgaria; 6 Centre for Advanced Studies in Electronics Science and Technology (CASEST), University of Hyderabad, Hyderabad 500046, Telangana, India

**Keywords:** hafnium, laser ablation in liquids, nanofibres, nanoparticles, nanostructures

## Abstract

This work presents a unique and straightforward method to synthesise hafnium oxide (HfO_2_) and hafnium carbide (HfC) nanoparticles (NPs) and to fabricate hafnium nanostructures (NSs) on a Hf surface. Ultrafast picosecond laser ablation of the Hf metal target was performed in three different liquid media, namely, deionised water (DW), toluene, and anisole, to fabricate HfO_2_ and HfC NPs along with Hf NSs. Spherical HfO_2_ NPs and nanofibres were formed when Hf was ablated in DW. Hf ablated in toluene and anisole demonstrated the formation of core–shell NPs of HfC with a graphitic shell. All NPs exhibited novel optical reflectance properties. Reflectance measurements revealed that the fabricated NPs had a very high and broad optical absorption throughout the UV–vis–NIR range. The NPs synthesised in toluene exhibited the best absorption. The successful fabrication of Hf NSs with the formation of laser-induced periodic surface structures (LIPSS) with low spatial frequency (LSFL) and high spatial frequency (HSFL) orthogonal to each other was also demonstrated. The LSFL and HSFL both exhibited quasi-periodicity. This work presents a simple way to fabricate HfO_2_ and HfC NPs and provides insight into their morphological and optical characteristics paving way for their applications in future.

## Introduction

Hafnium (Hf) is a tetravalent transition metal with compounds showing excellent thermal and optical properties [1–4]. Hf and its alloys are used in nuclear reactors because of their large neutron absorption cross sections and high melting points [5]. They are also used in submarines because of their corrosion resistance [6–7]. The high refractoriness of some Hf compounds [2,4] allows them to be used in high-temperature alloys and ceramics. Hf compounds are widely used in microelectronics because of their high dielectricity values [2]. In recent years, NPs derived from Hf have gained significant interest in biomedical fields because of their superior optical and thermal properties [8] compared to bulk Hf. HfO_2_ is a wide-bandgap (5.68 eV) material with a high dielectric constant (≈25) [9–10]. HfC has a very high melting point (≈3900 °C) and ranks among the hardest materials, with a Vickers hardness value exceeding 20 GPa [4,11]. The properties vary substantially depending on size and morphology [1]. Recently, interest has risen regarding synthesis and study of Hf-based NPs. Depending on the morphology, chemical composition, and quantum confinement effects, NPs can exhibit novel properties, making them applicable for large-spectrum usage [12–13]. Thus, synthesizing the desired morphology is essential for a given application. Generally, practical techniques for obtaining nanomaterials are sol–gel method, chemical and physical vapour deposition, hydrothermal method, ball milling, grinding, lithography, etching, and laser ablation [14–18]. The morphology determines the electrical and optical properties, which can vary depending on the synthesis technique [19]. Among the methods mentioned above, laser ablation in liquids (LAL) is a clean and single-step synthesis method used for obtaining nanomaterials from a bulk source [11,16–18,20]. It produces NPs of high purity with minimal or no unwanted by-products [11,17,21], thus making it a valuable candidate for green synthesis [21–22].

In the LAL method, a high-energy ultrashort pulsed laser (nanosecond, picosecond, or femtosecond) is focused on the surface of the target material immersed in a liquid medium. The target material absorbs the pulse energy via the electrons. It transfers it to the lattice, which expulses the surface material as a plasma plume confined because of the pressure created by the surrounding liquid [16,20,23–24]. A cavitation bubble is formed as the energy is transferred to the surrounding liquid from the decaying plasma because of the existing temperature differences between the liquid and the plasma plume, leading to the emergence of a vapour layer with a volume equivalent to the plasma plume [16,20,23–24]. The cavitation bubble collapses because of cyclic expansion and shrinkage, releasing nanoparticles into the surrounding liquid. The formed nanoparticles stay in the liquid as colloidal suspensions or can agglomerate to form a precipitate [6,16,20,23–27]. LAL provides flexibility regarding the choice of the liquid medium surrounding the target, from a single pure medium to a mixture of liquid media, with a range of target types such as powder, pellets, and well-defined structures and shapes [16,20]. The choice of the liquid medium can significantly affect morphology and chemical composition of the obtained NPs. The high energy of the laser pulses sometimes causes a reaction between the surrounding liquid medium and ablated target molecules, which may lead to the formation of unusual or non-equilibrium nanodimensional products [25–26,28–29]. Only few works on the laser ablation of Hf in liquid media have been reported in the literature. In our earlier reports, HfO_2_ nanoparticles, nanoribbons, and nanofibres were synthesised by ablating HfO_2_ pellets utilising femtosecond laser pulses at 800 nm [10,30]. A bulk Hf target was also ablated in another work using nanosecond laser pulses in different liquids to synthesise oxides and carbides [11,31]. In one of our earlier works [10], we performed femtosecond ablation and reported the formation of colloidal hafnium oxide NPs and nanoribbons in deionised water. The average sizes of NPs and nanoribbons were 13.5–18.0 and 10–20 nm, respectively. Further, we also reported that monoclinic and hexagonal phases were observed at higher input pulse energies. We believe these correspond to HfO_2_ and Hf_6_O, respectively. Further, in [10], we did not explore the formation of surface nanostructures on Hf after ablation. Therefore, the current study intends to understand the role of input pulse duration (picosecond pulses used here) and the surrounding liquid medium on the laser-ablated Hf-based NPs and NSs. Three different solvents, deionised water (DW; inorganic and oxygen-containing), toluene (organic and oxygen-free), and anisole (organic and oxygen-containing), have been chosen as ablation media. The Hf target was ablated with a picosecond laser in these three solvents to make three different colloidal solutions of Hf-based NPs. The optical, morphological, and physical properties of the obtained Hf-based NPs were studied in detail. The morphology of the ablated Hf surface in the three liquids was also investigated.

## Experimental

### Materials

**Figure 1:**
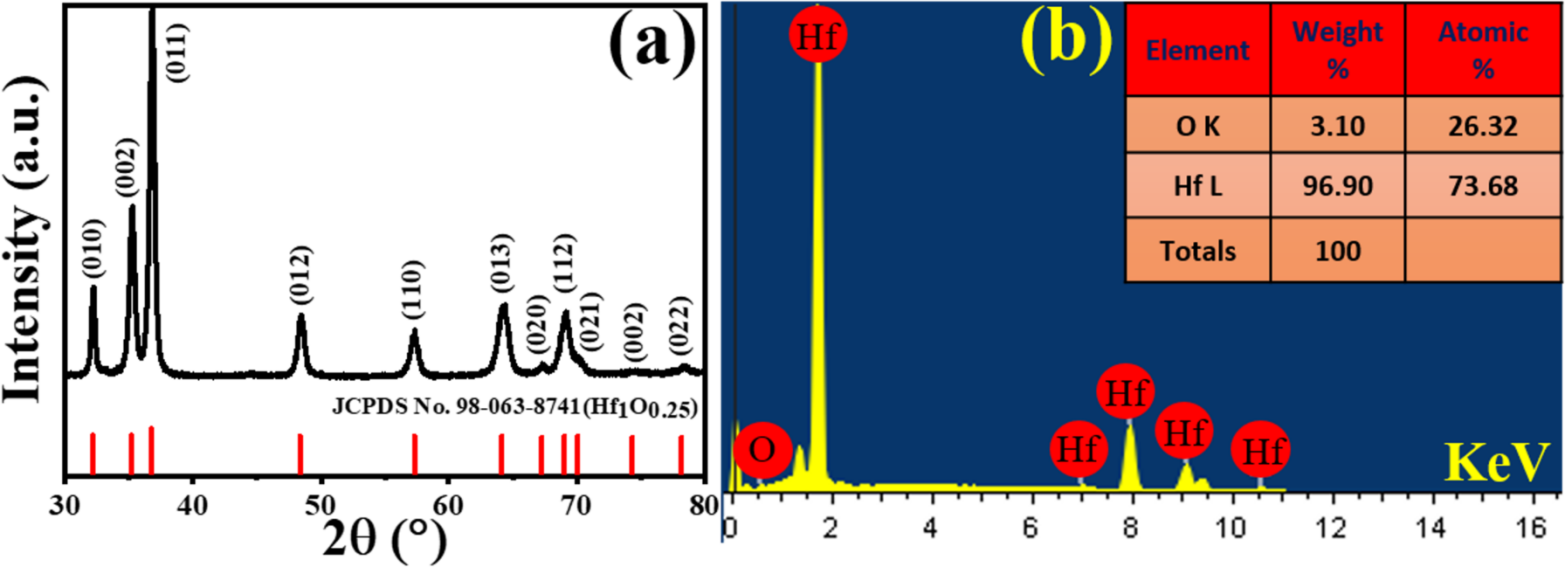
(a) XRD data and (b) EDX data of pristine Hf target.

Hf sponge was produced by metallurgical operations involving solvent extraction, briquetting, carbochlorination, Kroll reduction and vacuum distillation. The sponge samples were further refined by consolidation and refining under vacuum (3–6 × 10^−5^ mbar) using an electron beam melting furnace having a beam power of 60 kW (ELIT 60) at an accelerating voltage of 24 kV in a water-cooled crucible with feeding mechanism and an extraction system [32]. All operations were conducted at the Centre for Materials for Electronics Technology (CMET), Hyderabad. These Hf sponges, cut and polished to 10 mm × 10 mm × 2 mm, were used as ablation targets. The pristine target had the crystal structure of hexagonal HfO_0.25_, as confirmed by X-ray diffraction (XRD) data (see Figure 1a). The elemental composition (Hf: 73.68%, O: 26.32%) was determined by energy-dispersive X-ray spectroscopy (EDX, Figure 1b). Distilled water with a resistivity of more than 18 MΩ·cm was obtained from a Millipore system. Toluene and anisole (spectroscopic grade) were obtained from Sigma-Aldrich and used as received.

### Synthesis of nanoparticles and nanostructures

**Figure 2:**
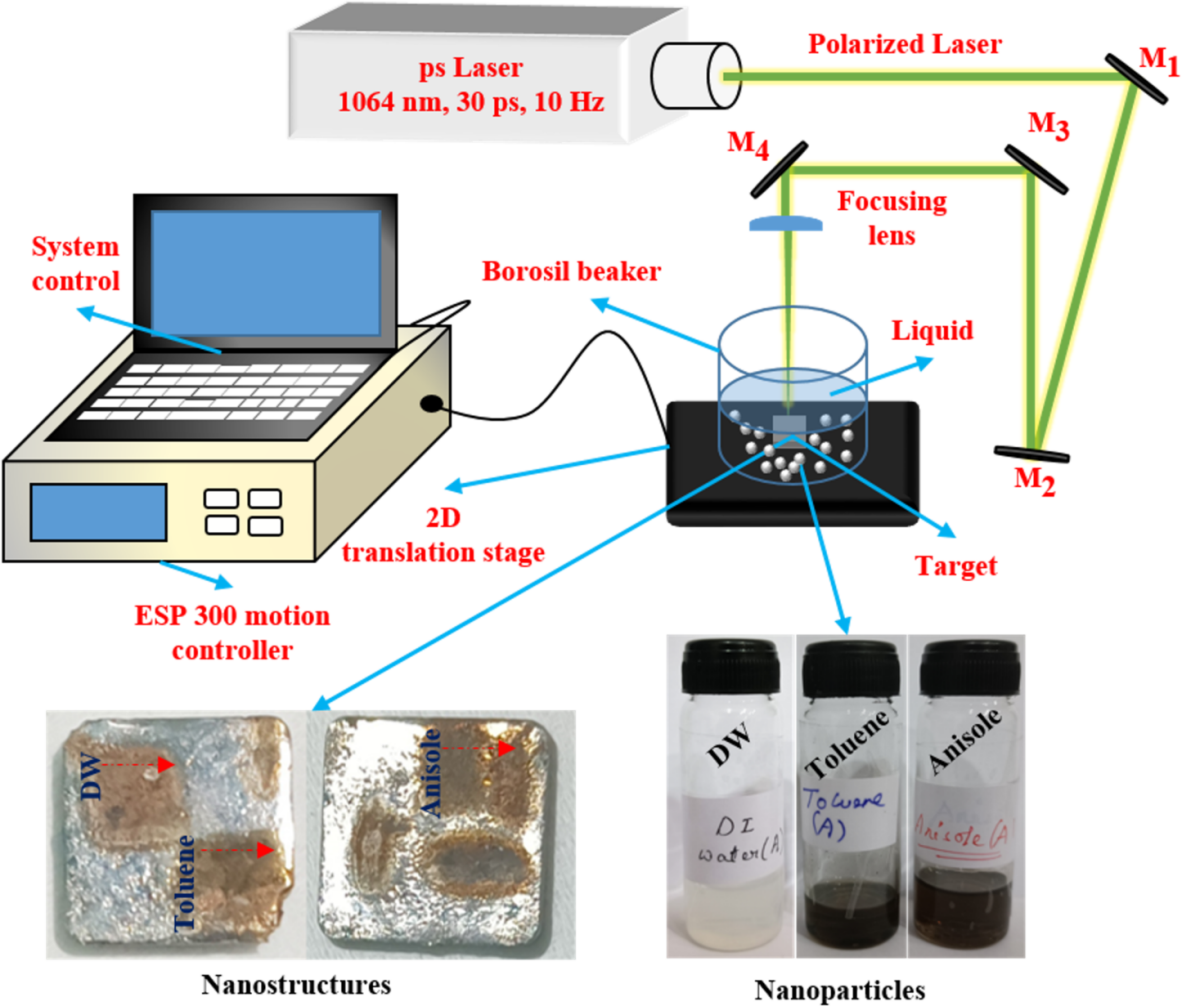
Schematic representation of the experimental setup used for picosecond LAL of a Hf target (*M*_n_ represents mirrors).

A linearly polarised picosecond laser [Nd:YAG, M/s EKSPLA] with a pulse duration of ≈30 ps, a repetition rate of ≈10 Hz, a wavelength of ≈1064 nm, and a pulse energy of ≈16.3 mJ (determined from previous experiments [33] and optimised using multiple ablation trials followed by detailed characterization studies) was used for the fabrication of the NPs and NSs. The ablation was performed in three different liquids, that is, DW, toluene, and anisole. As illustrated in Figure 2, the incoming laser beam was focused vertically on the Hf target in a liquid-filled glass cell using a plano-convex lens (*f* = 80 mm). The liquid surface was about 5 mm above the target surface. Raster scanning was performed at a speed of 0.1 mm/s to ablate an area of 5 × 5 mm^2^. This resulted in Hf surface nanostructures and Hf NPs forming in the surrounding liquid. A gradual colour change of the liquids initially confirmed the formation of Hf-based NPs; DW turned from transparent to turbid white, while toluene and anisole turned from transparent to black (Figure 2). The obtained NPs and NSs were labelled as described in Table 1, according to the liquid in which they were ablated, and subsequently characterised through different methods.

**Table 1:** Labelling of the NPs and NSs according to the liquid media used.

Liquids used	NPs	NSs

DW	HfNPs-D	HfNSs-D
toluene	HfNPs-T	HfNSs-T
anisole	HfNPs-A	HfNSs-A

### Characterization techniques

The synthesised NPs were drop-cast on carbon-coated copper grids to record transmission electron microscopy (TEM) images and selected area electron diffraction (SAED) patterns using a FEI Tecnai G2 S-Twin operating at 200 kV. Further, these NPs were drop-cast on cleaned Si substrates, and their morphology was analysed using field-emission scanning electron microscopy (FESEM); the composition was determined by EDX attached to the FESEM (Carl Zeiss Smart SEM ULTRA 55). Reflectivity was investigated using a UV–vis–NIR spectrometer (PerkinElmer Lambda 750). For photoluminescence (PL) measurements, a Horiba LabRAM HR Evolution (Excitation: 325 nm, Lens: 40×, spot size: 1 μm) was used. Image J software was used to extract spatial periodicities and to generate 2D fast Fourier transform images (2D FFT) of the Hf surface structures.

## Results and Discussion

### Nanoparticles

**Figure 3:**
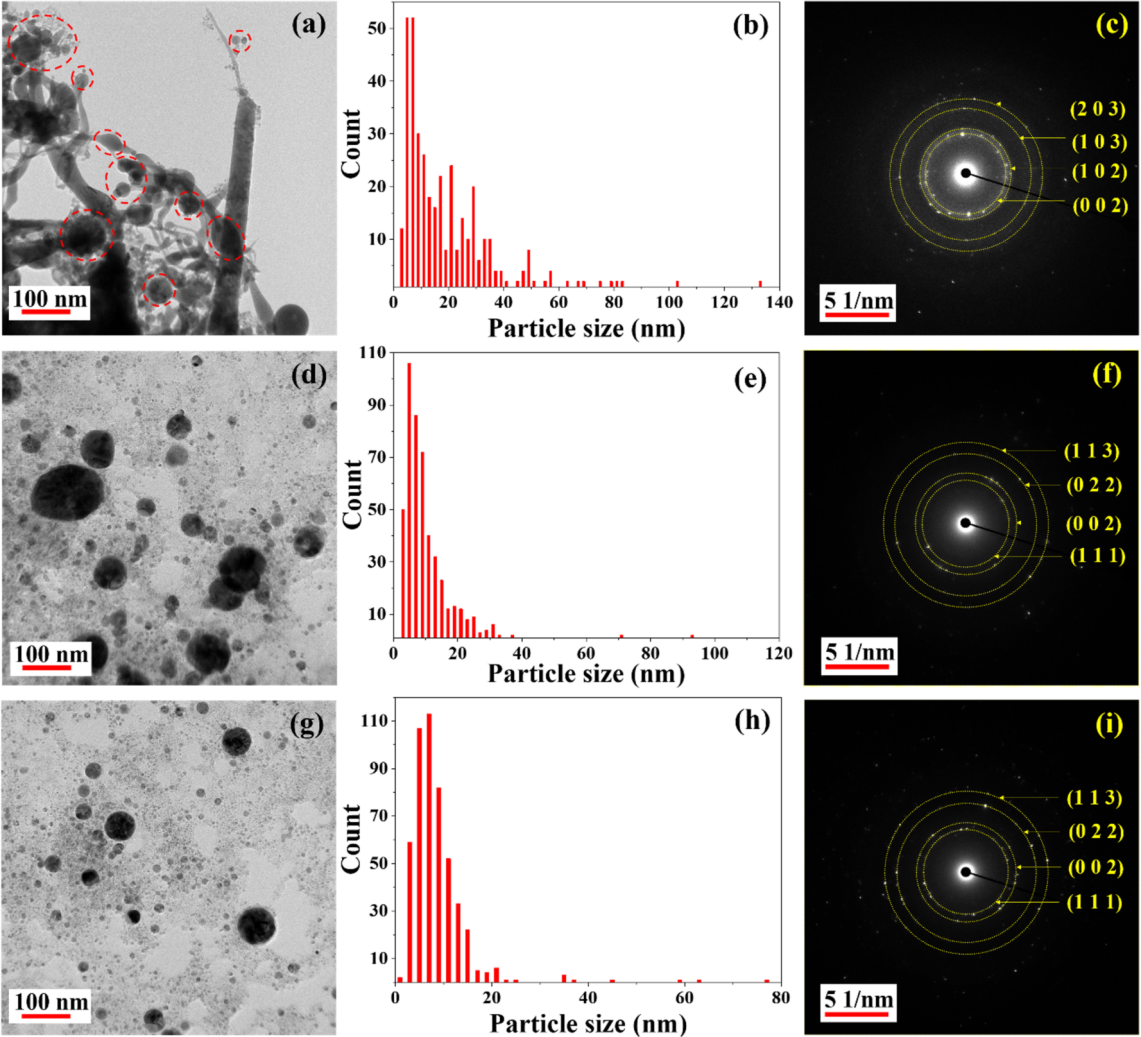
TEM images, particle size distributions, and SAED patterns of laser-ablated NPs in (a–c) DW, (d–f) toluene, and (g–i) anisole.

Figure 3 shows TEM images, the corresponding particle size distributions, and the SAED patterns of NPs obtained in DW (Figure 3a–c), toluene (Figure 3d–f), and anisole (Figure 3g–i). The TEM image corresponding to HfNPs in DW shows the formation of nanofibres of diameters ranging from 5 to 65 nm along with spherical NPs (marked with red dashed circles, Figure 3a). The formation of nanofibres is consistent with our earlier observations for HfO_2_ ablation in DW [10,30]. Further, the TEM images corresponding to Hf NPs in toluene and anisole (Figure 3d,g) illustrate the formation of spherical particles only. The majority of the NPs had a size distribution in the ranges of 5–40 nm in DW and 5–20 nm in toluene and anisole, respectively, as shown in Figure 3b, Figure 3e, and Figure 3h, respectively. The SAED patterns shown in Figure 3c,f,i indicate that the NPs were polycrystalline. The planes shown in Figure 3c for HfNPs-D were found to be (203), (103), (102), and (002) corresponding to orthogonal hafnium oxide HfO_2_ (ICDD: 98-008-7456). In contrast, HfNPs-T (Figure 3f) and HfNPs-A (Figure 3i) exhibited the (111), (002), (022), and (113) planes corresponding to hafnium carbide HfC [ICDD: 98-018-5992]. The observation of HfO_2_ in DW and HfC in toluene and anisole can be attributed to chemical interactions between the ablated Hf atoms and the liquid medium.

**Figure 4:**
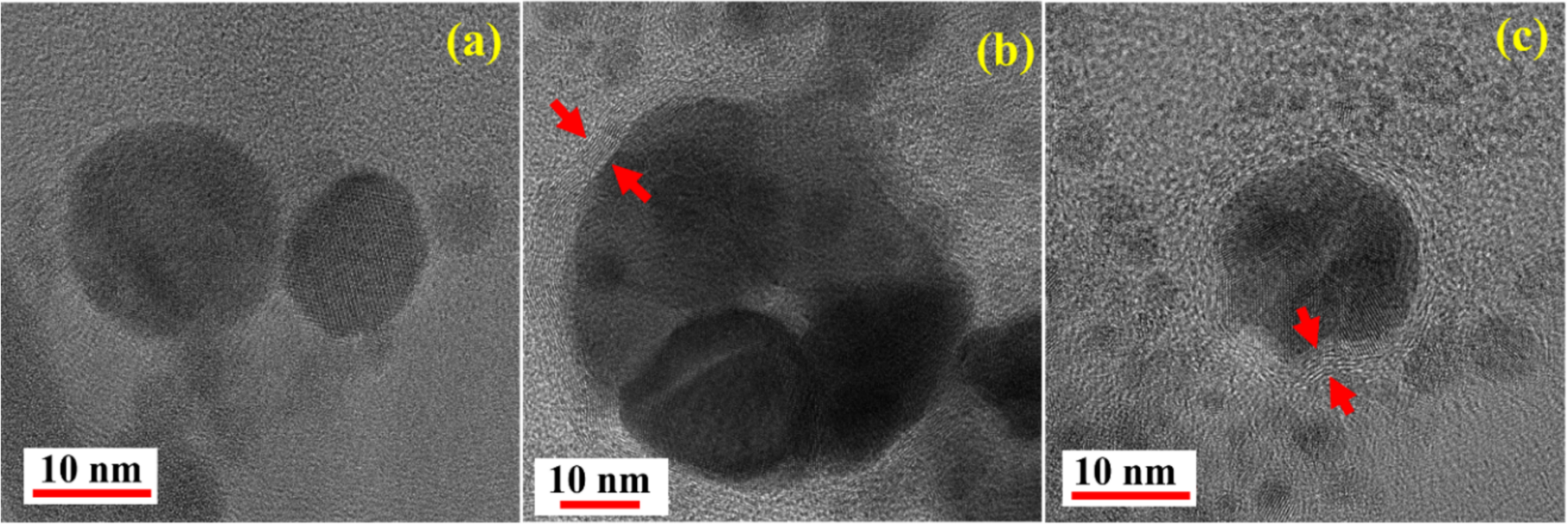
High-resolution TEM images of laser-ablated NPs in (a) DW, (b) toluene, and (c) anisole.

**Figure 5:**
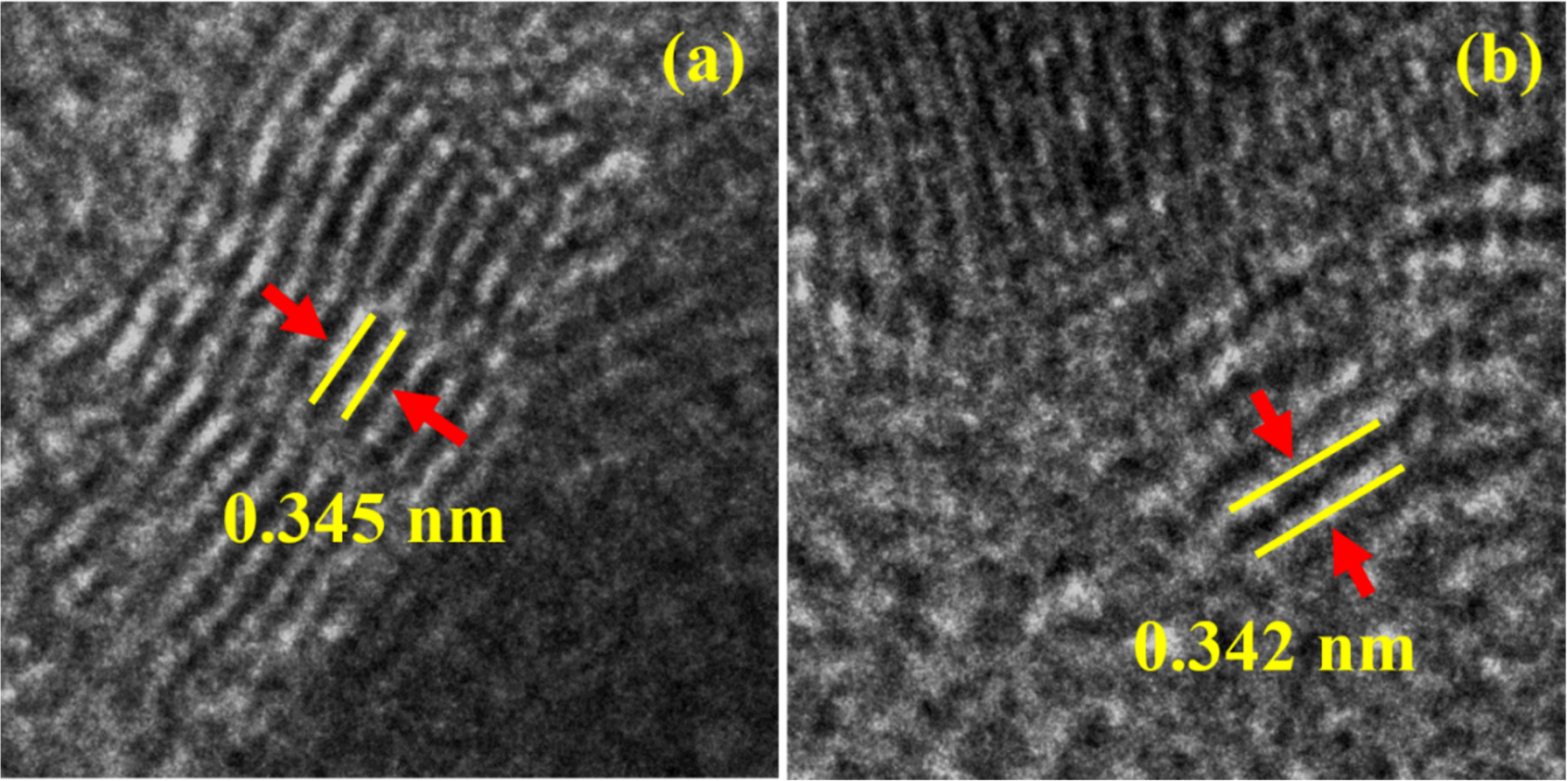
Zoomed TEM images of the shell-like structures shown in Figure 4b and Figure 4c for (a) toluene and (b) anisole (the d-spacings of the outer shells are indicated with yellow lines).

Careful observation of high-resolution TEM images revealed the formation of core–shell structures for the particles obtained in anisole and toluene (Figure 4b,c). In contrast, we did not notice such a structure in the case of NPs fabricated in DW (Figure 4a). Shell-like structures in Figure 4b and Figure 4c are indicated with red arrows. These structures are multilayered carbon shells around the NPs. Similar formations were noticed in other studies where carbon-rich liquids were used [34–36]. The d-spacing of the carbon shells was determined from the zoomed images in Figure 5. It was estimated to be ≈0.34 nm, confirming the outer shell to be made of graphite [37–39].

**Figure 6:**
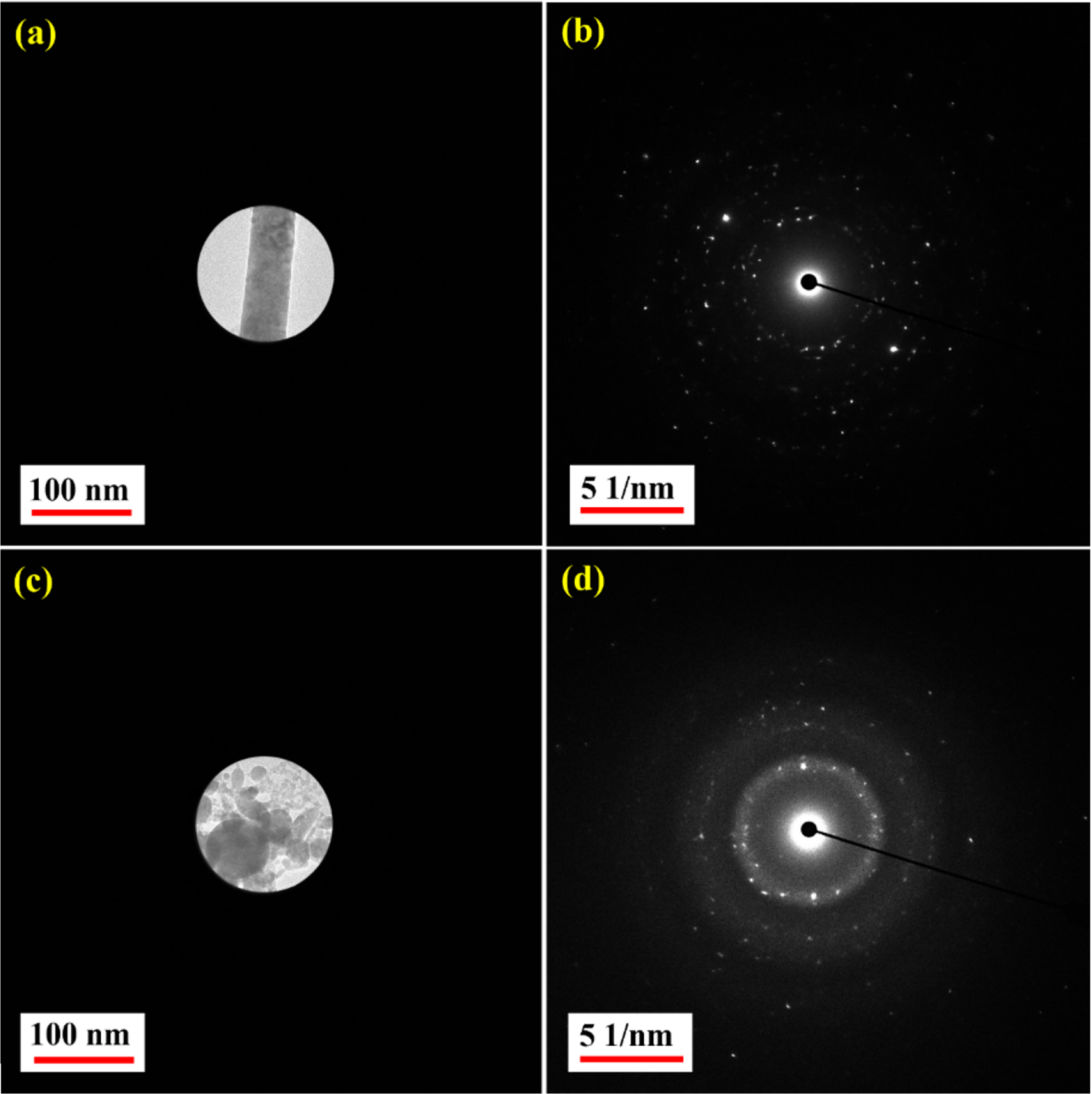
SAED measurement locations and patterns for (a, b) nanofibres and (c, d) NPs obtained in DW.

A similar analysis was conducted on the nanofibre-like structures formed when Hf was ablated in DW. Figure 6 shows nanofibres (Figure 6a) and NPs (Figure 6c) formed in DW, together with the corresponding SAED patterns (Figure 6b and Figure 6d, respectively). A difference can be seen between the crystallinity of the nanofibres and that of the NPs. Figure 6b shows that the nanofibres are perfectly polycrystalline. In contrast, in Figure 6d, the presence of diffused rings for HfNPs-D indicates a mix of amorphous and polycrystalline phases in the NPs. The formation of HfO_2_ NPs in DW along with nanofibres and the observed crystallinity patterns can be explained by considering the decomposition of the surrounding H_2_O molecules due to the laser energy [23–24,40]. This leads to the reaction of oxygen with Hf^4+^ ions in the plasma plume formed during the ablation [16,20,23–24,41], leading to the formation of hafnium oxide vapour as the plasma decays. As the pressure of the surrounding liquid exceeds the vapour pressure exerted by HfO_2_, the cavitation bubble collapses, and the vapour rushes through the liquid in the form of a jet [23–24,41]. The lower temperature of the surrounding liquid leads to the formation of nuclei [23,42–43] with random crystallographic orientation, which grow to form crystals [43–45]. These crystals coalesce to form a polycrystalline structure [43,46]. As the vapour rushes out as a jet, these polycrystals assemble [43,47] linearly to form nanofibres. The formation of these nanofibres seems to depend upon laser parameters such as pulse duration, repetition rate, wavelength, and pulse energy, as is evident from earlier reports [11,18,31], where no such nanofibre formation is reported. However, the formation of nanofibres has been reported in [10,30]. Due to Brownian motion [48], some nuclei and crystals escape from the jet flow and agglomerate [43,47–48], leading to the formation of nanoparticles [43]. Some of these nuclei with abundant hafnium oxide molecules around grow like crystals [45,48]. The agglomeration [47] of such crystals leads to the observed polycrystalline state in the NPs [43,46]. The formation of the observed amorphous NPs is due to the suppression of nucleation [43,49]. Thus, a mix of amorphous and polycrystalline structures in NPs obtained in DW is seen. The formation of these HfO_2_ NPs and nanofibres is responsible for the turbid white colour observed after ablation in DW.

Earlier reports on Hf ablation in toluene [11,31] did not discuss the formation of graphite shells around HfC NPs. The formation of polycrystalline HfC core–shell NPs with graphite shells similar to [36] in toluene and anisole can be explained by the possible reaction of carbon from decomposed surrounding liquid with Hf^4+^ ions in the plasma plume [20,23–24,40]. As the plasma plume decays, its space is occupied by HfC vapour. The pressure difference due to the surrounding liquid causes the cavitation bubble to collapse; thus, the vapour rushes through the liquid and forms polycrystals, similar to the above case of HfO_2_ [16,20,23–24,41,43]. The decomposed surrounding liquid has a carbon-rich environment. As the polycrystals grow, they also act as a nucleus for carbon atoms to self-assemble [27,50], forming graphite layers and, eventually, core–shell NPs of different sizes. This formation of graphite layers might prevent the formation of fibres. As no hafnium oxide was observed, it can be stated that oxygen in the plasma from HfO_0.25_ did not react with Hf^4+^. This can be explained through the fact that the carbon-rich environment near the plasma shifts the reaction equilibrium in favour of the formation of HfC [23–24,51–52]. Thus, the O^2+^ ions do not react with the Hf^4+^ ions. The O^2+^ ions can react with C or escape the liquid as O_2_. The black colour observed in toluene and anisole after ablation is due to the formation of HfC core–shell NPs and the decomposition of the surrounding liquid [27,39,53]. The formation of carbides by LAL in aromatic solvents was reported previously with transition metals such as iron and cobalt [54–56]. Kanitz et al. [55] have reported the formation of pure (i.e., with a clean surface) iron nanoparticles when the target was ablated with femtosecond pulses (5 kHz repetition rate, though) in different solvents. They observed that the choice of the surrounding liquid environment allowed them to tune the properties of the iron-based NPs, for example, the generation of iron oxides or carbides. In the present case, the surrounding liquid possibly had a huge influence on the formation of carbides. For a given Hf target (regarding purity and surface roughness), the ablation products depend critically on the surrounding liquid, input pulse duration, input pulse energy, and the number of pulses incident on the sample.

**Figure 7:**
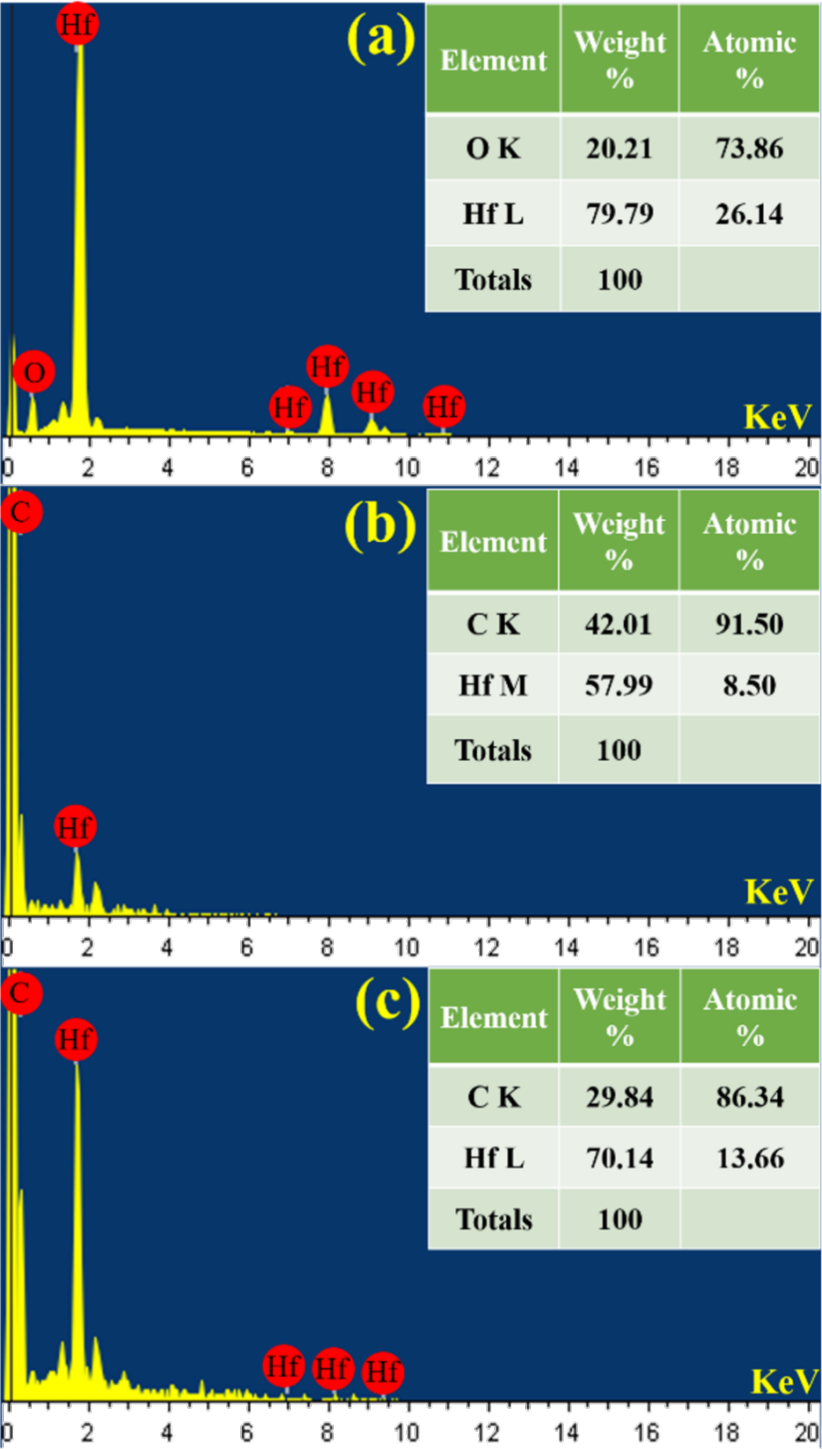
EDX spectra and elemental composition of (a) HfNPs-D, (b) HfNPs-T, and (c) HfNPs-A.

Figure 7 illustrates the EDX data for the particles synthesised in DW, toluene, and anisole. The EDX spectra of HfNPs-D confirm the presence of hafnium and oxygen (Figure 7a). The observed atomic percentages are 73.86 atom % oxygen and 26 atom % Hf (Figure 7a). The composition tables for HfNPs-T (Figure 7b) and for HfNPs-A (Figure 7c) show the presence of carbon and hafnium. The high fraction of C indicates the formation of the graphitic shell around HfC NPs in both toluene and anisole.

**Figure 8:**
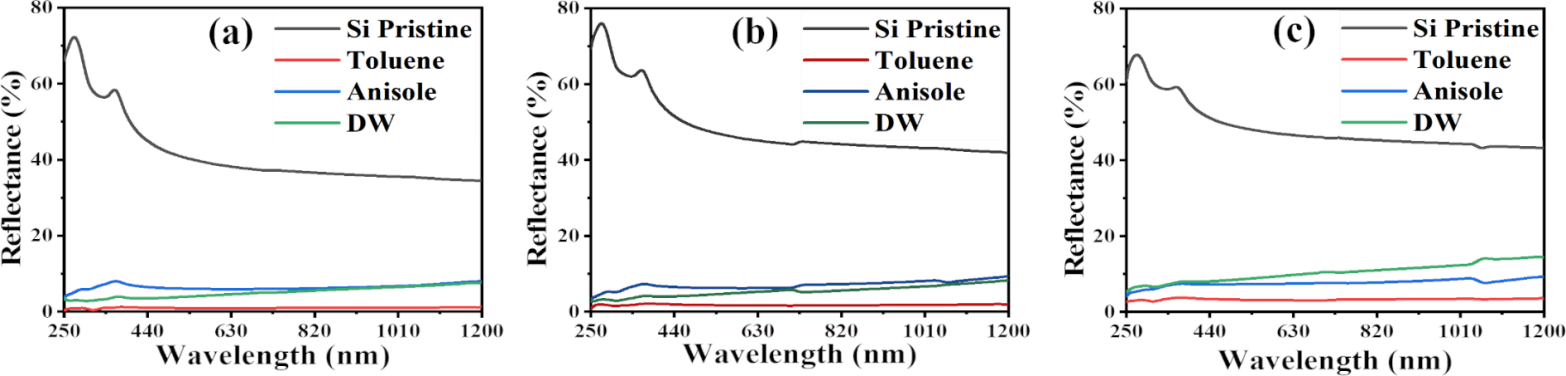
Reflectance spectra of NPs drop-cast on Si substrates measured under different incident angles: (a) 30°, (b) 45°, and (c) 60°. The black curve is the reflectance spectrum of the reference Si substrate; the red, blue, and green curves represent Hf NPs synthesised in in toluene, anisole, and DW, respectively.

Figure 8 shows the reflectance data of a pristine Si substrate compared to a Si substrate coated with HfNPs-D, HfNPs-T, and HfNPs-A under three different angles of incidence (30°, 45°, and 60°) taken in the wavelength range from 250 to 1200 nm. The black curve corresponds to the reflectance spectrum of the reference pristine Si sample; the red curve is HfNPs-T, the blue curve is HfNPs-A, and the green curve is HfNPs-D. The values of the reflectance and reduction in the UV (λ = 250 nm) and the NIR (λ = 1200 nm) spectral regions of the NPs under different angles of incidence are summarised in Table 2. Based on the data, it can be concluded that Hf NPs show a very high and wide optical absorption from UV to NIR. HfNPs-T especially show exceptional performance compared to other NPs with far superior and stable optical absorption compared to similar HfC NPs synthesised in our earlier work [31]. With an increase in angle, a reduction in absorption was observed for HfNPs-D, HfNPs-T, and HfNPs-A. A decrease in absorption was also observed with an increase in wavelength at a constant angle. The deviation in the spectral pattern of HfNPs-T and HfNPs-A could be due to the presence of oxygen in the polycrystalline lattice of HfNPs-A. The additional oxygen in anisole compared to toluene might have been included in the NPs’ polycrystalline structure during NP formation. We can confirm that the oxygen from anisole has not reacted with the Hf^4+^ ions as no oxide compound was found in the SAED data of HfNPs-A (Figure 3i) as compared to HfNPs-T (Figure 3f). Thus, the extra oxygen in anisole is likely to be present as an impurity in the polycrystalline structure of the NPs in HfNPs-A and may affect the optical properties of the NPs.

**Table 2:** Reflectance and reduction in reflection in the UV and NIR regions of the NPs under different angles of incidence (θ).

θ		UV (λ = 250 nm)	NIR (λ = 1200 nm)

		reflectance (%) (reduction of reflectance)	reflectance (%) (reduction of reflectance)

30°	Si (pristine)	66.71% (–)	34.56% (–)
	HfNPs-D	3.35% (94.97%)	7.83% (77.34%)
	HfNPs-T	0.72% (98.92%)	1.11% (96.78%)
	HfNPs-A	3.35% (94.97%)	7.83% (77.34%)

45°	Si (pristine)	70.26% (–)	41.94% (–)
	HfNPs-D	3.28% (95.33%)	8.23% (80.37%)
	HfNPs-T	0.92% (98.69%)	1.87% (95.54%)
	HfNPs-A	3.28% (95.33%)	9.41% (77.56%)

60°	Si (pristine)	61.43% (–)	43.39% (–)
	HfNPs-D	5.45% (91.12%)	14.55% (66.46%)
	HfNPs-T	2.81% (95.42%)	3.66% (91.56%)
	HfNPs-A	4.27% (93.05%)	9.55% (77.99%)

**Figure 9:**
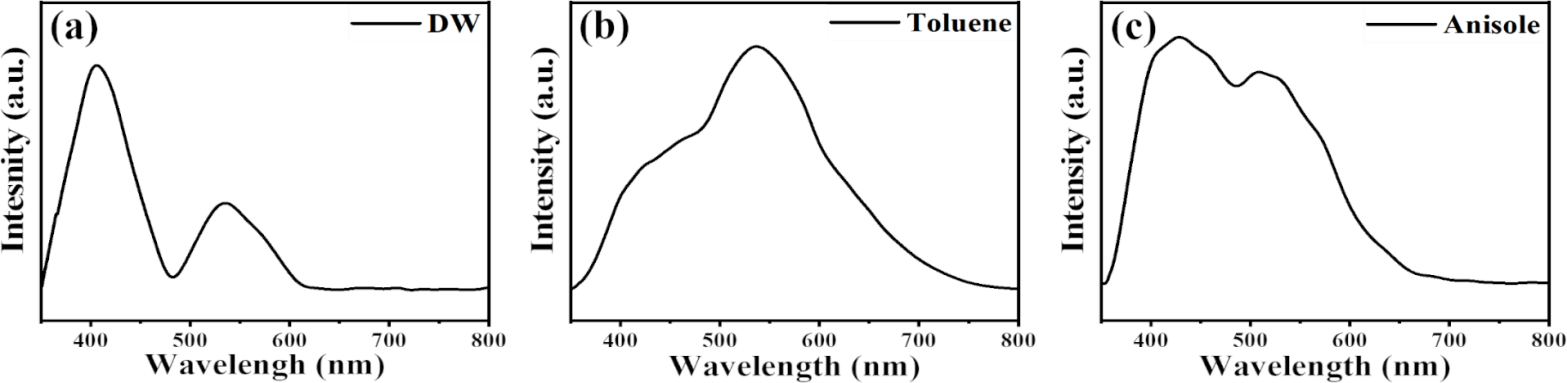
PL spectra of NPs laser-ablated in (a) DW, (b) toluene, and (c) anisole.

Figure 9a–c shows the PL emission spectra of the NPs laser-ablated in DW, toluene, and anisole, respectively. Emission peaks were observed for each of the NPs (Figure 9). The presence of emission peaks indicates the presence of defects in the NPs [57]. The defects may be due to impurities in the lattice structure, possibly in the form of oxygen contamination, or imperfect crystallinity of the graphitic layer or the NPs themselves. Further detailed PL studies are essential to understand the origin of the observed emission peaks.

### Nanostructures

**Figure 10:**
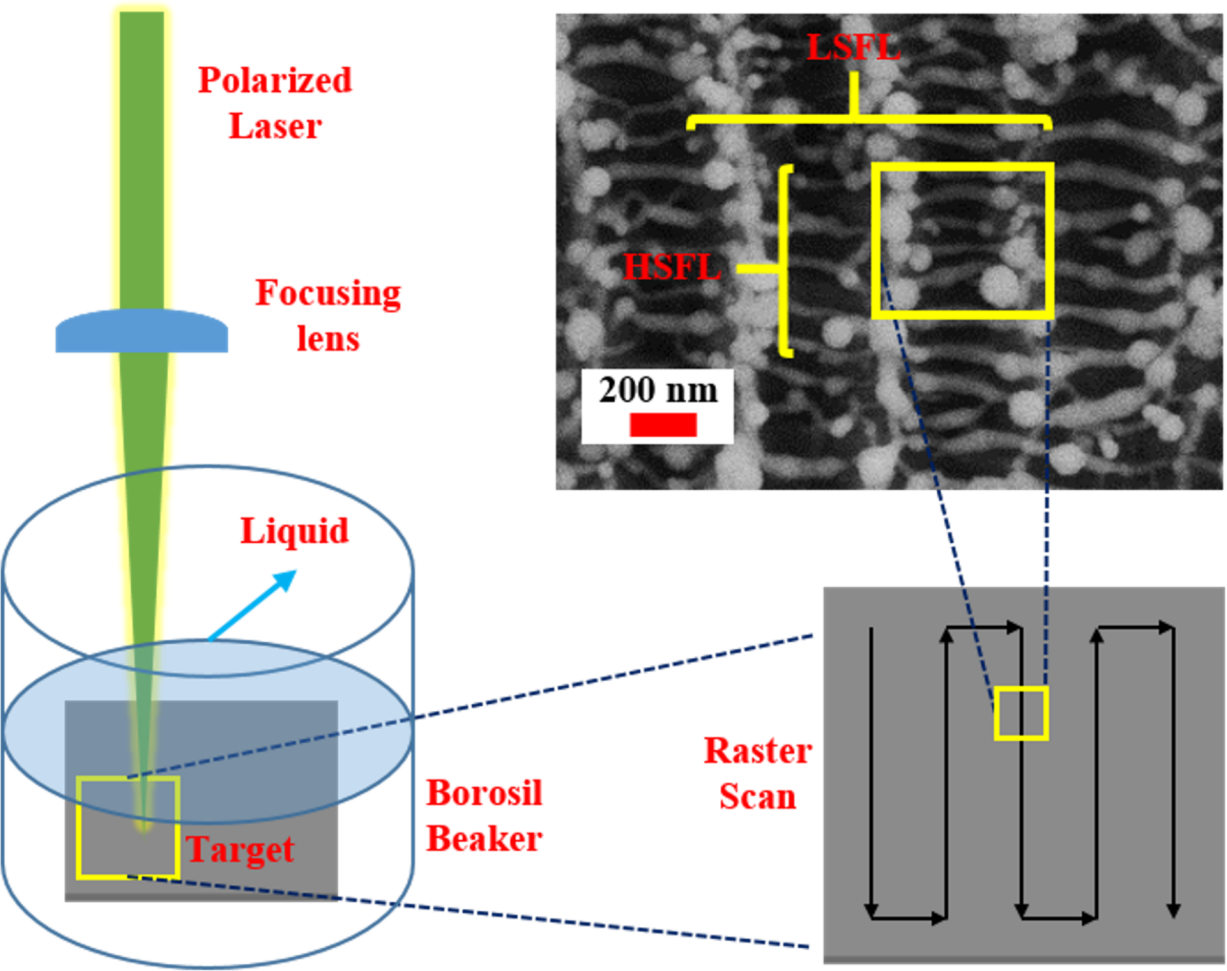
Schematic of the NS fabrication by raster scanning the sample, resulting in LSFL and HSFL formation.

Figure 10 illustrates the NS fabrication with picosecond LAL by raster scanning the Hf target. The figure also depicts the LSFL and HSFL formed on the target during the scanning process. The LAL technique is versatile since the NPs and NSs are obtained simultaneously in a single experiment, which is impossible with any other lithographic technique (laser-based or otherwise). However, the patterning of the target will influence the obtained NPs and NSs since the number of pulses incident on a particular surface area will vary with different scanning/writing conditions. Scanning parameters (e.g., speed of the stage or spot size of the laser) can be varied to achieve an optimum size of the NPs and NSs. The simultaneous formation of HSFL and LSFL on the Hf target during LAL was observed in all liquids. The LSFL structures were oriented parallel to the laser scanning direction, and the HSFL structures were formed in the depressions of the LSFL with a direction perpendicular to the direction of the laser scan.

**Figure 11:**
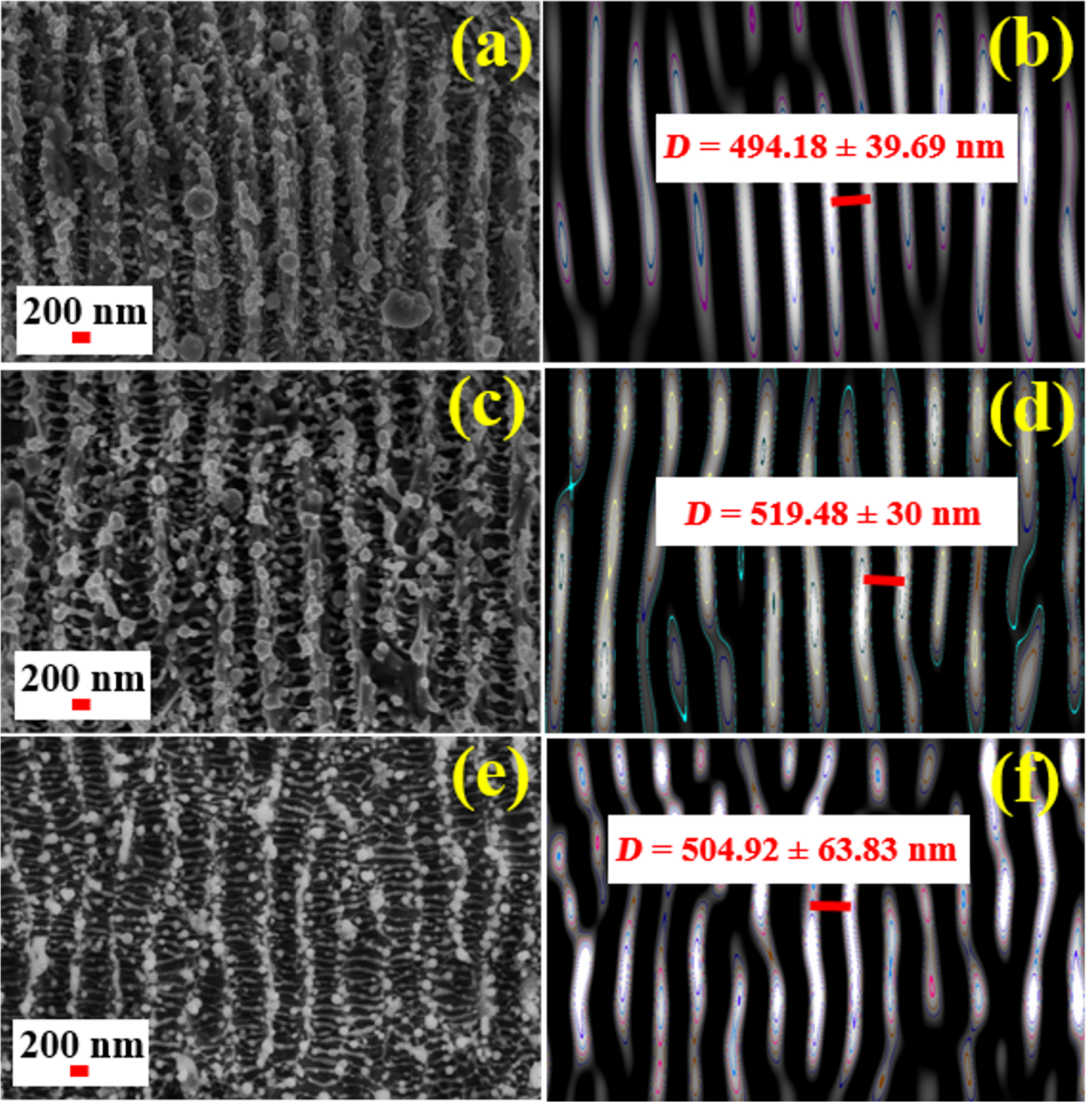
FESEM image and inverse FFTs of LSFL with spatial periodicity on laser-ablated NSs in (a, b) DW, (c, d) toluene, and (e, f) anisole.

**Figure 12:**
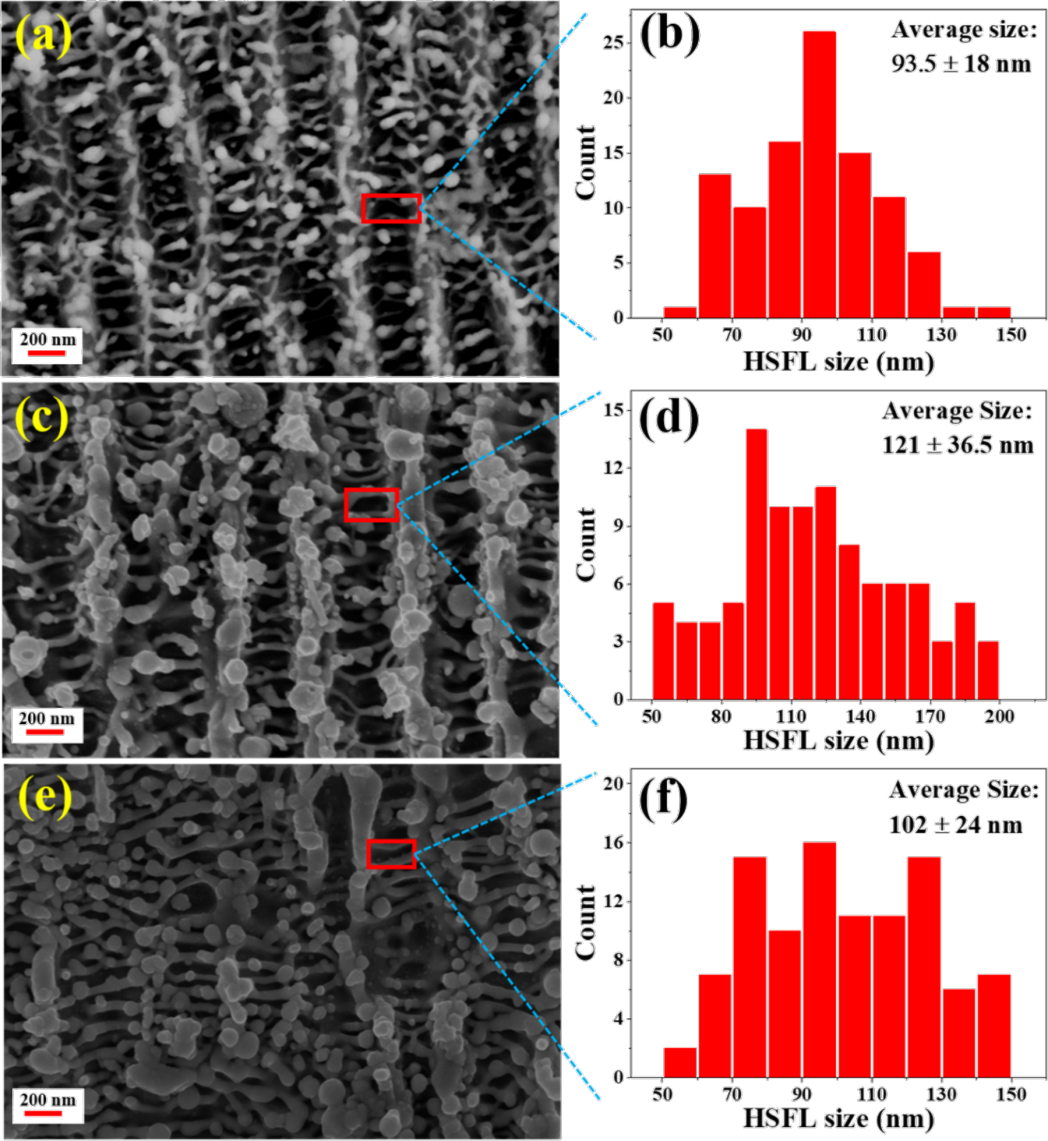
FESEM image and HSFL size distribution of NSs laser-ablated in (a, b) DW, (c, d) toluene, and (e, f) anisole.

Similar observations regarding the formation of LSFL and HSFL with orthogonal directionality and the plausible mechanisms behind their formation are discussed in an earlier work [58]. Figure 11 shows FESEM images of the laser-ablated NSs and 2D FFTs of LSFL with spatial periodicity for HfNSs-D, HfNSs-T, and HfNSs-A analysed using ImageJ software. Based on the data analysis, it can be concluded that the structures are quasi-periodic and have a sub-wavelength periodicity of λ_L_/2 or greater (λ_L_ is the laser wavelength). The quasi-periodicity values indicated as *D* in Figure 11b,d,f were 498 ± 40 nm for HfNSs-D, 519 ± 30 nm for HfNSs-T, and 505 ± 64 nm for HfNSs-A. On further inspection of the FESEM images of the NSs, the formation of HSFL was observed. Figure 12 shows FESEM images of the HSFLs and the corresponding distribution of their feature size in HfNSs-D, HfNSs-T, and HfNSs-A.

The structures show sub-wavelength quasi-periodicity. The observed HSFL had an average feature size between λ_L_/11 and λ_L_/8 for all NSs. The feature size for HfNSs-D ranged from 50 to 150 nm, with an average feature size of 94 ± 18 nm, that of HfNSs-T ranged from 50 to 200 nm, with an average feature size of 121 ± 37 nm, and that of HfNSs-A ranged from 50 to 150 nm with an average feature size of 102 ± 24 nm.

**Figure 13:**
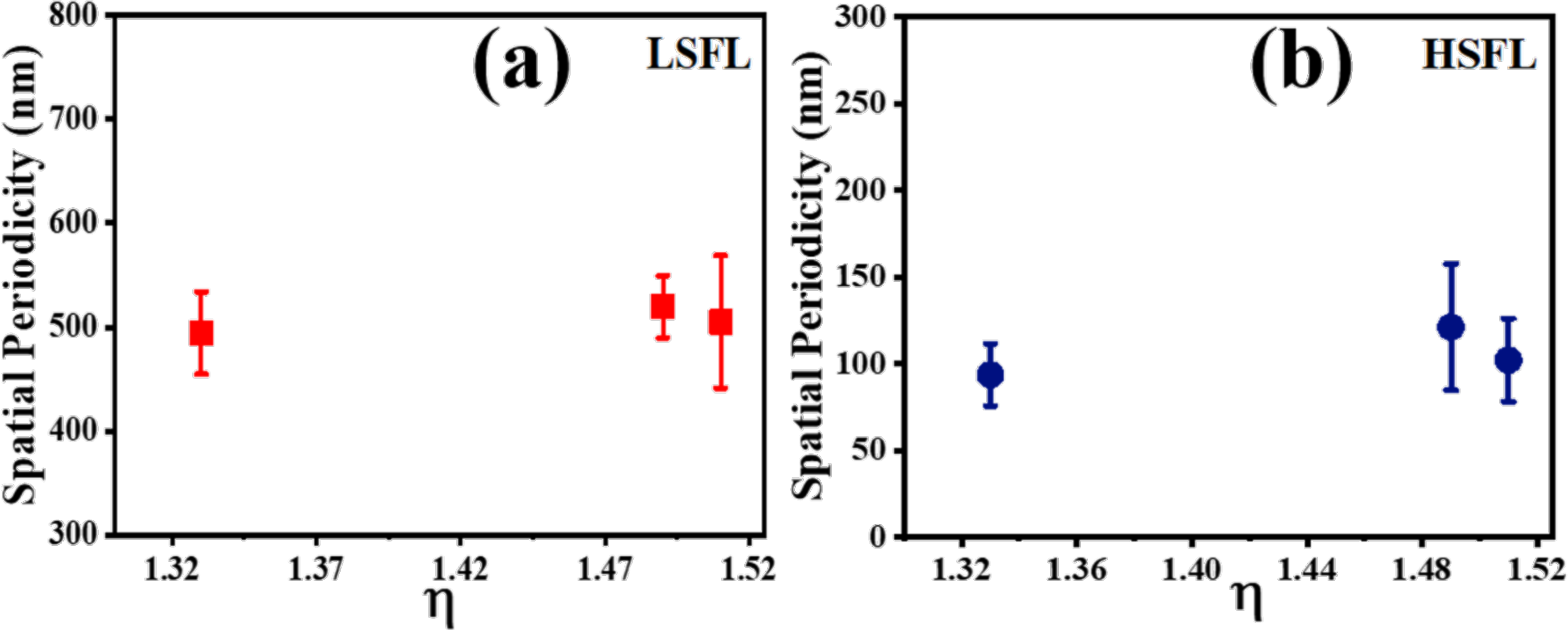
Spatial periodicity of (a) LSFL and (b) HSFL as function of the refractive index.

Figure 13 shows the relationship between the refractive index (η) of the liquid used during ablation and the corresponding spatial periodicity for LSFL and HSFL. The spatial periodicity was observed to increase from HfNSs-D (η^DW^ ≈ 1.33 [59]) to HfNSs-T (η^toluene^ ≈ 1.49 [60]) and to decrease again for HfNSs-A (η^anisole^ ≈ 1.51 [61–62]). The values are summarised in Table 3. The observable HFSL size appears to be independent on λ_L_ [63], instead the HSFL size depends on laser parameters such as fluence, energy dose, and pulse duration [64]. Re-deposition and re-solidification of the ablated NPs on NSs were also observed, which matches with observation in an earlier reported work [65] for titanium ablation. These properties make Hf metal suitable for laser patterning of sub-wavelength-size structures, and the choice of the liquid for LAL enables the variation of feature size. We have used linearly polarised light in the present study. The orientation of the LIPSS depends on the polarization and rotates with the input polarization. The available literature on LIPSS suggests that there will be no changes in the spacing or other morphological features except the orientation of the LIPSS with respect to the polarisation. Combined with the picosecond laser’s high precision processing and industrial scalability, Hf is a potential material for sophisticated design patterning [66].

**Table 3:** Spatial periodicity of LSFL and HSFL in NSs as funcition of the refractive index of the liquid used for ablation.

Liquids used	Refractive index (η)	NSs	LSFL (nm)	HSFL (nm)

DW	≈1.33	HfNSs-D	498 ± 40	94 ± 18
toluene	≈1.49	HfNSs-T	519 ± 30	121 ± 37
anisole	≈1.51	HfNSs-A	505 ± 64	102 ± 24

## Conclusion

The current study shows the successful single-step fabrication of HfO_2_ NPs and nanofibres in DW and HfC core–shell NPs with multilayered graphitic shells in toluene and anisole via LAL of Hf metal. The obtained NPs exhibit a broad size distribution. Most NPs had a diameter between 5 and 20 nm. The HfNPs-D, HfNPs-T, and HfNPs-A NPs were found to be polycrystalline. The oxygen in anisole was found to be inert during ablation and was possibly incorporated as impurity in HfNPs-A. The HfO_2_ nanofibres were also found to be polycrystalline, with diameters ranging from 5 to 65 nm. The NPs showed very high and broad optical absorption throughout the UV–vis–NIR range. The maximum absorption was observed at 30° at UV (λ = 250 nm) for HfNPs-T with just 0.72% reflection. The absorption decreases in HfNPs-D, HfNPs-T, and HfNPs-A with an increase in wavelength and angle of incidence. The NPs are suitable for application in optical devices requiring high and stable optical absorption throughout the UV–vis–NIR range. The successful fabrication of Hf NSs with the formation of LIPSS, LSFL and orthogonal HSFL, was also demonstrated. The LSFL and HSFL both showed quasi-periodicity. The spatial periodicity of LSFL and HSFL first increased and then decreased with respect to the refractive index of the liquid used during ablation. This form of data is highly valuable to optimise the feature sizes during laser patterning in standard ablation liquids. The study will be extended to other metallic targets and more liquids to create a more comprehensive report.

## Data Availability

Data generated and analyzed during this study is available from the corresponding author upon reasonable request.
